# Gamma Knife radiosurgery with CT image‐based dose calculation

**DOI:** 10.1120/jacmp.v16i6.5530

**Published:** 2015-11-08

**Authors:** Andy (Yuanguang) Xu, Jagdish Bhatnagar, Greg Bednarz, Ajay Niranjan, Douglas Kondziolka, John Flickinger, L. Dade Lunsford, M. Saiful Huq

**Affiliations:** ^1^ Department of Radiation Oncology University of Pittsburgh Cancer Institute Pittsburgh PA; ^2^ Department of Neurological Surgery University of Pittsburgh Medical Center Pittsburgh PA; ^3^ Department of Neurosurgery New York University Medical Center New York NY USA

**Keywords:** Gamma Knife, dose calculation, inhomogeneity effect

## Abstract

The Leksell GammaPlan software version 10 introduces a CT image‐based segmentation tool for automatic skull definition and a convolution dose calculation algorithm for tissue inhomogeneity correction. The purpose of this work was to evaluate the impact of these new approaches on routine clinical Gamma Knife treatment planning. Sixty‐five patients who underwent CT image‐guided Gamma Knife radiosurgeries at the University of Pittsburgh Medical Center in recent years were retrospectively investigated. The diagnoses for these cases include trigeminal neuralgia, meningioma, acoustic neuroma, AVM, glioma, and benign and metastatic brain tumors. Dose calculations were performed for each patient with the same dose prescriptions and the same shot arrangements using three different approaches: 1) TMR 10 dose calculation with imaging skull definition; 2) convolution dose calculation with imaging skull definition; 3) TMR 10 dose calculation with conventional measurement‐based skull definition. For each treatment matrix, the total treatment time, the target coverage index, the selectivity index, the gradient index, and a set of dose statistics parameters were compared between the three calculations. The dose statistics parameters investigated include the prescription isodose volume, the 12 Gy isodose volume, the minimum, maximum and mean doses on the treatment targets, and the critical structures under consideration. The difference between the convolution and the TMR 10 dose calculations for the 104 treatment matrices were found to vary with the patient anatomy, location of the treatment shots, and the tissue inhomogeneities around the treatment target. An average difference of 8.4% was observed for the total treatment times between the convolution and the TMR algorithms. The maximum differences in the treatment times, the prescription isodose volumes, the 12 Gy isodose volumes, the target coverage indices, the selectivity indices, and the gradient indices from the convolution and the TMR 10 calculations are 14.9%, 16.4%, 11.1%, 16.8, 6.9%, and 11.4%, respectively. The maximum differences in the minimum and the mean target doses between the two calculation algorithms are 8.1% and 4.2% of the corresponding prescription doses. The maximum differences in the maximum and the mean doses for the critical structures between the two calculation algorithms are 1.3 Gy and 0.7 Gy. The results from the two skull definition methods with the TMR 10 algorithm agree either within ± 2.5% or 0.3 Gy for the dose values, except for a 4.9% difference in the treatment times for a lower cerebellar lesion. The imaging skull definition method does not affect Gamma Knife dose calculation considerably when compared to the conventional measurement‐based skull definition method, except in some extreme cases. Large differences were observed between the TMR 10 and the convolution calculation method for the same dose prescription and the same shot arrangements, indicating that the implementation of the convolution algorithm in routine clinical use might be desirable for optimal dose calculation results.

PACS numbers: 87.55.D, 87.55.kd

## I. INTRODUCTION

The TMR dose calculation algorithm and the 24‐point measurement‐based manual skull definition method have been the basis of the Gamma Knife radiosurgery treatment planning for many years.^1^, ^2^ The manual skull definition method is a convenient approach for modeling the shape of a human head for dose calculation.^(3)^ The water‐based TMR algorithm provides a fast dose calculation method for real‐time interactive treatment planning in Gamma Knife radiosurgery.

Many studies have been done to evaluate the performance of the TMR dose calculation algorithm and the manual skull definition method.^3^, ^4^, ^5^, ^6^, ^7^, ^8^, ^9^, ^10^, ^11^ The TMR algorithm is demonstrated to work better for targets located at the center of the brain than for those in the peripheral and/or heterogeneous regions.^2^, ^4^, ^5^, ^6^, ^7^, ^8^, ^9^ The manual skull definition method is in general considered a good approximation owing to the fact that the Gamma Knife dose calculation is relatively insensitive to the local variations in the skull contour.^3^, ^10^, ^11^

In recent years, a CT image‐based automatic skull definition method and a convolution dose calculation algorithm were developed for the Leksell GammaPlan software version 10 (Elekta, Stockholm, Sweden).^(12)^ The imaging skull definition method provides an option to delineate patient skull shape based on automatic segmentation of the whole head CT images. The convolution algorithm introduces tissue inhomogeneity correction to the dose calculation in Gamma Knife treatment planning for the first time.^13^, ^14^, ^15^, ^16^

The implementation of the two new approaches for routine clinical use is still a subject of discussion at many institutions,^(17)^ even though technically there is not much difficulty for doing this. Many questions remain to be answered about these new approaches including: i) Is it worthwhile to acquire whole head CT images for all patients for the purpose of more accurate skull definition? ii) Is it necessary to replace the TMR algorithm by the convolution algorithm for dose calculation in Gamma Knife radiosurgery? and iii) Is the dose prescription guideline for the TMR algorithm valid for the convolution algorithm?

The purpose of this work was to conduct a comparative study on a set of clinical treatment plans to evaluate the potential impact of the imaging skull definition method and the convolution dose calculation algorithm on the Gamma Knife radiosurgery treatment planning.

## II. MATERIALS AND METHODS

Sixty‐five patients who underwent CT image‐guided Gamma Knife radiosurgeries at the University of Pittsburgh Medical Center in recent years were retrospectively studied. CT images were obtained instead of MRI images for these patients because of the presence of surgical or dental implants in the patient body. Some of the patients treated with CT image‐guided dose planning were not included in the study because the imaging skull definition method could not be applied successfully. Either the top of the skull or the edges of the stereotactic frame were not included in the CT images for these patients. Out of the 65 patients studied, 39 were treated on a Gamma Knife model Perfexion unit and 26 on a Gamma Knife model 4C unit (Eletka). The total numbers of treatment targets on the Perfexion and the 4C units are 69 and 35, respectively. The diagnoses for these cases include trigeminal neuralgia (TGN), meningioma, acoustic neuroma, arteriovenous malformation (AVM), glioma, and benign and metastatic brain tumors. Table 1 gives a list of the number of patients, the number of treatment targets, and the range of the prescription doses for each disease group.

**Figure 1:**
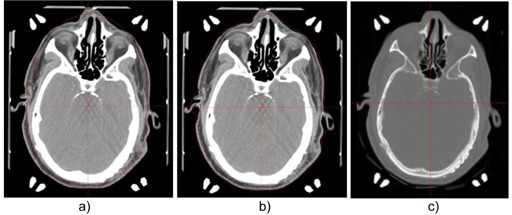
A sample transverse CT slice with the skull contours from the manual skull definition method (a) and the imaging skull definition method (b). The electron density map for the slice is shown in (c).

After the CT images for a patient were imported into the planning system, a CT image‐based skull shape and a 3D electron density map were generated for the patient. The upper and the lower thresholds for the automatic segmentation tool in the GammaPlan were set at 2000 and −200 Hounsfield units. The resolution for the segmentation was set at 0.5 mm. Manual editing of the skull contours was found necessary for some patients, usually in the lower neck region around the stereotactic base frame. Figure 1 shows a sample CT slice with the skull contours from the imaging and the manual skull definition methods, along with the electron density map for this slice.

Dose calculations were performed for each patient using the convolution and the TMR 10 algorithms with the same prescription doses, the same prescribed isodose lines, and the same shot arrangements. For each treatment matrix, 11 parameters including the total treatment time (TT), the prescription isodose volume (PIV), the 12 Gy isodose volume (V12),^18^, ^19^ the target volume (TV) receiving the prescription dose (${\text{PIV}}_{TV}$), the isodose volume of the half prescription dose (${\text{PIV}}_{50\%}$), the minimum, maximum and the mean doses on the treatment target, and the critical structures under consideration were recorded. The patient skull geometry was then changed to the manual definition and a TMR 10 calculation was performed for the same plan.

The three sets of data recorded were processed using Microsoft excel spreadsheets. The target coverage index ($\text{TCI}={\text{PIV}}_{50\%}/\text{TV}$), the selectivity index ($\text{SI}={\text{PIV}}_{50\%}/\text{PIV}$),^(20)^ and the gradient index ($\text{GI}={\text{PIV}}_{50\%}/\text{PIV}$)^(21)^ were calculated for each treatment target. These indices are often used in Gamma Knife radiosurgery to evaluate a treatment plan in terms of the coverage of the treatment target, the conformity of the planned dose distribution, and the falloff of the radiation doses beyond the treatment area. To evaluate the tissue inhomogeneity effect, the ratios of the total treatment times, the prescription isodose volumes, the 12 Gy isodose volumes, the target coverage indices, the selectivity indices, and the gradient indices from the convolution algorithm and the TMR 10 algorithm with imaging skull definition were calculated for each matrix, along with the differences in the minimum target doses (${\text{TD}}_{\text{min}}$), the mean target doses (${\text{TD}}_{\text{mean}}$), the maximum critical structure doses (${\text{SD}}_{\text{max}}$), and the mean critical structure doses (${\text{SD}}_{\text{mean}}$). To compare the results from the two skull definition methods, the procedure was repeated for the two TMR 10 calculations.

**Table 1:** Number of patients, number of treatment matrices, and the range of prescription dose for each disease group

	*TGN*	*Acoustic*	*AVM*	*Meningioma*	*Benign*	*Glioma*	*Mets*	*Total*
Patients	10	3	4	14	5	8	21	65
Matrices	10	3	4	17	6	12	52	104
Doses (Gy)	35–42.5	12.5–14	16–18	12–15	11–14	13–16	14–20	

## III. RESULTS

**Figure 2:**
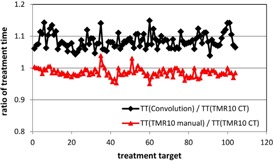
Ratios of the treatment times from the three dose calculation approaches for the 104 treatment matrices. Black line = ratio between the convolution calculation and the TMR 10 calculation with imaging skull definition; red line = ratio between the TMR 10 calculation with manual skull definition and the TMR 10 calculation with imaging skull definition.

Figure 2 compares the ratios of the total treatment times from the three dose calculation approaches using the convolution algorithm and the TMR 10 algorithm with two different skull definition methods. The treatment time from the convolution calculation is on the average 8.4% higher than that from the TMR 10 calculation with image skull definition for the 104 treatment matrices studied. The maximum difference between the two calculations was found to be 14.9% for a treatment target in the frontal region of the skull. Similar results were reported from previous studies on model human head CT images in which a maximum decrease of 11.5% in delivered dose was observed for treatment targets in the superior frontal/parietal vertex region owing to the excessive radiation attenuation of the skull bone.^(17)^

The differences in the treatment times between the convolution and the TMR 10 algorithms can be attributed to the combination effects of all the bone/air inhomogeneities along the pathways of individual radiation beams. For treatment shots placed close to the patient skull, the attenuation depths of the radiation beams from certain directions are small and the percentages of the attenuation depths in the skull bone are high. The contribution from the high radiation attenuations in the dense bone in these beams plays an important role in the overall dose calculation process.

The treatment time from the manual skull definition method is on the average 1.5% lower than that from the imaging skull definition method. The maximum difference in the treatment times from the two skull definition methods was found to be 4.9% for the treatment of a cerebellar lesion in a patient with an irregularity on the neck. In this case, the imaging skull definition method picks up the irregularity on the skull contour, whereas the manual skull definition makes a straight line extrapolation.

**Figure 3:**
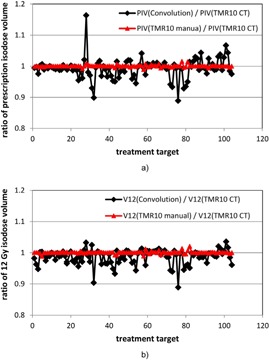
Ratios of the prescription isodose volumes (a) and the 12 Gy isodose volumes (b) from the three dose calculation approaches for the 104 treatment matrices. Black line = ratio between the convolution calculation and the TMR 10 calculation with imaging skull definition; red line = ratio between the TMR 10 calculation with manual skull definition and the TMR 10 calculation with imaging skull definition.

Figure 3 shows the ratios of the prescription isodose volumes and the 12 Gy isodose volumes from the three calculation approaches. The maximum differences between the convolution algorithm and the TMR 10 algorithm were found to be 16.4% and 11.1% for the prescription isodose volume and the 12 Gy isodose volume, respectively. These numbers were obtained from two matrices that overlap with other matrices. A 10.1% difference in the prescription isodose volume and a 9.7% difference in the 12 Gy isodose volume were also observed for a treatment target near the sphenoid sinus and the sigmoid sinus, where pronounced tissue inhomogeneity effect is expected. The prescription isodose volumes and the 12 Gy isodose volumes from the TMR 10 calculations with two different skull definition methods all agree within ± 2%.

**Figure 4:**
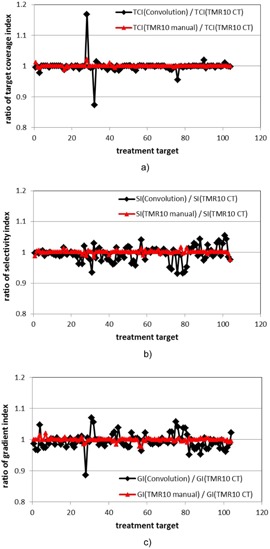
Ratios of the target coverage indices (a), the selectivity indices (b), and the gradient indices (c) from the three dose calculation approaches for the 104 treatment matrices. Black line = ratio between the convolution calculation and the TMR 10 calculation with imaging skull definition; red line = ratio between the TMR 10 calculation with manual skull definition and the TMR 10 calculation with imaging skull definition.

Figure 4 compares the target coverage indices, the selectivity indices and the gradient indices from the three calculation approaches for the 104 treatment matrices. The maximum differences between the target coverage indices, the selectivity indices, and the gradient indices from the convolution and the TMR 10 calculations are 16.8%, 6.9% and 11.5%, respectively. The differences in the target coverages are small for most of the cases because of the margins around the major portions of these treatment targets. The few exceptions are for highly conformal treatment plans with nearby treatment matrices or bone/air inhomogeneity. For both the selectivity indices and the gradient indices, the ratios between the results from the convolution and the TMR 10 algorithms are in general smaller than the corresponding values for the prescription isodose volume. This indicates that the differences in the prescription isodose volumes (PIV) may be partially offset by the differences in the target volumes receiving the prescription doses (${\text{PIV}}_{50\%}$) in the selectivity index calculation and the volumes of the half prescription doses (${\text{PIV}}_{50\%}$) in the gradient index calculation. No difference of more than 2.5% was observed for the results between the two TMR 10 calculations for all matrices.

**Figure 5:**
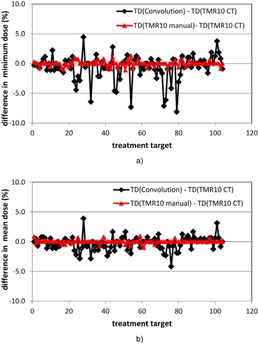
Differences in the minimum target doses (a) and the mean target doses (b) from the three dose calculation approaches for the 104 treatment matrices. Black line = difference between the convolution calculation and the TMR 10 calculation with imaging skull definition; red line = difference between the TMR 10 calculation with manual skull definition and the TMR 10 calculation with imaging skull definition. All the target dose (TD) differences are percent dose differences relative to the corresponding prescription isodoses.

Figure 5 shows the differences in the minimum target doses and the mean target doses for all the treatment targets resulting from the use of different calculation approaches. The maximum differences in the minimum and the mean target doses between the convolution algorithm and the TMR 10 algorithm are 8.1% and 4.2% of the corresponding prescription doses. More than 5% dose differences were observed between the minimum target doses from the two algorithms for five meningioma and metastasis cases. The maximum differences in the minimum target doses and the mean target doses from the two TMR algorithms were found to be 1.33% and 1%, respectively.

**Figure 6:**
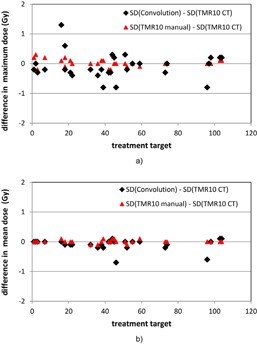
Differences in the maximum doses (a) and the mean doses (b) to the critical structures from the three dose calculation approaches for the 104 treatment matrices. Black line = difference between the convolution calculation and the TMR 10 calculation with imaging skull definition; red line = difference between the TMR 10 calculation with manual skull definition and the TMR 10 calculation with imaging skull definition.

Figure 6 shows the results for the differences in maximum doses and the mean doses on the critical structures under consideration resulting from the use of the three calculation approaches. Thirty critical structures, including the brainstems for the trigeminal neuralgia cases, the cochleae for the acoustic cases, and the optical structures for some of the meningioma and metastasis cases, were considered for the 65 patients. A difference of 1.3 Gy was found for the maximum brainstem doses from the convolution and the TMR 10 calculations for a trigeminal neuralgia patient. More than 0.5 Gy difference were also recorded for some of the maximum cochlea doses and the maximum optical structure doses. Except for two special cases, the mean doses on the critical structures from the convolution and the TMR 10 calculations agree within 0.3 Gy. All the results from the two TMR calculations with different skull definition methods agree within 0.3 Gy.

Tables 2 and 3 give the ranges of the 10 set of parameters presented in Fig. 2 to 6 for each disease group. Generally speaking, the parameters for the centrally located treatment targets in the trigeminal neuralgia and the acoustic neuroma cases change in much narrower ranges than those for the meningioma and metastases treatment targets, which could be located in all regions of a brain. This is consistent with the previous study on model patient in which the dose difference between the convolution and the TMR 10 algorithms was analyzed in term of the location of the treatment shots.^(17)^

It should be pointed out that the two isodose volumes, the three plan evaluation indices, and the four dose statistic parameters were obtained in parallel with the treatment time for each treatment target. The dosimetric differences between the three calculation approaches should be viewed as the combination effect of all these parameters.

**Table 2:** Comparisons of the dose statistics parameters from the convolution algorithm (series b) and the TMR 10 algorithm (series a) for each disease group. The target dose (TD) differences are percent dose differences relative to the prescription isodoses. The unit for the critical structure dose (SD) differences is Gy. The numbers in parentheses are negative

	*TGN*	*Acoustic*	*AVM*	*Meningioma*	*Benign*	*Glioma*	*Mets*
TT(b)T/T(a)	1.043–1.077	1.063–1.081	1.060–1.111	1.046–1.143	1.058–1.14	1.059–1.123	1.039–1.149
PIV(b)/PIV(a)	0.975–1.003	0.959–1.005	0.933–1.018	0.899–1.007	0.889–1.000	0.941–1.011	0.928–1.163
V12(b)/V12(a)	0.947–1.004	0.965–1.007	0.939–1.007	0.904–1.005	0.889–0.997	0.943–1.013	0.945–1.038
TCI(b)/TCI(a)	0.978–1.000	0.988–0.997	0.988–1.002	0.873–1.001	0.955–1.000	0.986–1.014	0.993–1.168
SI(b)/SI(a)	0.992–1.015	0.964–1.008	0.945–1.016	0.958–1.030	0.931–1.000	0.952–1.011	0.932–1.055
GI(b)/GI(a)	0.966–0.995	0.988–1.022	0.977–1.024	0.975–1.058	0.992–1.041	0.985–1.038	0.886–1.037
${\text{TD}}_{\text{min}}(b)-{\text{TD}}_{\text{min}}(a)$	(1.18)–1.18	(0.91)–1.43	(6.15)–2.78	(7.33)–0.83	(4.17)–0.71	(7.14)–0.83	(8.12)–4.44
${\text{TD}}_{\text{mean}}(b)-{\text{TD}}_{\text{mean}}(a)$	(0.71)–0.71	(1.43)–0.00	(2.31)–1.67	(2.86)–0.77	(4.17)–0.71	(2.14)–0.77	(2.86)–3.89
${\text{SD}}_{\text{max}}(b)-{\text{SD}}_{\text{max}}(a)$	(0.40)–1.30	(0.80)–0.00	(0.30)–0.30	(0.80)–0.20	(0.30)	(0.40)–0.20	0.00–0.20
${\text{SD}}_{\text{mean}}(b)-{\text{SD}}_{\text{mean}}(a)$	(0.10)–0.00	(0.20)–0.10	(0.20)–0.10	(0.70)–0.10	0.00	(0.20)–0.00	0.00–0.10

**Table 3:** Comparisons of the dose statistics parameters from the manual skull definition approach (series c) and the imaging skull definition approach (series a) for each disease group. The target dose (TD) differences are percent dose differences relative to the prescription isodoses. The unit for the critical structure dose (SD) differences is Gy. The numbers in parentheses are negative

	*TGN*	*Acoustic*	*AVM*	*Meningioma*	*Benign*	*Glioma*	*Mets*
TT(c)/TT(a)	0.976–1.003	0.985–1.001	0.980–1.030	0.957–1.008	0.953–1.000	0.972–1.010	0.951–1.039
PIV(c)/PIV(a)	0.999–1.001	1.000–1.003	0.997–1.007	0.997–1.008	0.995–1.001	0.989–1.008	0.992–1.016
V12(c)/V12(a)	0.999–1.004	1.000–1.003	0.993–1.000	0.988–1.012	0.993–1.002	0.989–1.008	0.994–1.021
TCI(c)/TCI(a)	0.988–1.012	0.997–1.000	0.999–1.000	0.998–1.010	0.994–1.000	1.000–1.001	0.998–1.022
SI(c)/SI(a)	0.987–1.012	0.999–1.000	0.997–1.007	0.984–1.007	1.000–1.001	0.989–1.008	0.976–1.015
GI(c)/GI(a)	0.999–1.020	0.994–1.000	0.984–1.003	0.992–1.015	0.994–1.003	0.996–1.004	0.977–1.008
${\text{TD}}_{\text{min}}(c)-{\text{TD}}_{\text{min}}(a)$	(0.71)–0.29	(0.91)–0.8	(1.11)–1.11	(0.83)–1.33	(0.91)–0.00	(0.77)–0.83	(1.00)–1.00
${\text{TD}}_{\text{mean}}(c)-{\text{TD}}_{\text{mean}}(a)$	(0.50)–0.86	0.00–0.80	(0.56)–0.00	(1.00)–0.95	(0.71)–0.00	0.00–0.01	(0.50)–0.71
${\text{SD}}_{\text{max}}(c)-{\text{SD}}_{\text{max}}(a)$	(0.20)–0.30	(0.10)–0.10	0.00–0.30	(0.10)–0.10	(0.20)	0.00	0.00–0.10
${\text{SD}}_{\text{mean}}(c)-{\text{SD}}_{\text{mean}}(a)$	0.00–0.10	(0.10)–0.10	0.00–0.10	(0.10)–0.10	0.00	(0.10)–0.10	0.00

## IV. DISCUSSION

Dose calculations for Gamma Knife radiosurgery depend on a precise characterization of the treatment unit, an accurate definition of the patient skull geometry, and a reliable algorithm for modeling the tissue–radiation interaction. With the introduction of the new treatment planning system in recent years, the CT image‐based skull definition method and the convolution dose calculation algorithm became available for routine clinical use.^12^, ^13^, ^14^, ^15^, ^16^ The pros and cons of these new approaches need to be evaluated before they can be implemented for routine clinical use.

The 24‐point, measurement‐based skull definition method has been used for simulating the patient skull geometry since the installation of the first Gamma Knife unit in the United States.^(1)^ The CT image‐based skull definition method is certainly a more accurate alternative to the manual skull definition method in that it can reproduce the fine structures on the patient skull surface. In this study, small dosimetric differences (less than 2.5% or 0.3 Gy) were observed for most of the results between the two skull definition methods. The exceptions include the treatment of a lesion in the lower cerebellar region in a patient with an irregularity on the neck, for which a 4.9% difference in the treatment times was observed. Therefore, the limitations of the manual skull definition method need to be considered in some special cases and the use of the CT image‐based skull definition is a must for these cases.

The comparisons between the results from the convolution and the TMR 10 algorithms indicate that the implementation of the convolution algorithm in routine clinical use merits serious consideration. An average difference of 8.4% in the total treatment times from the two algorithms was observed for the same prescription dose and the same shot arrangements. For certain lesions in the frontal regions and/or around the air/bone inhomogeneities, the difference in the treatment times could be as much as 14.9%. A considerable underdose in the treatment planning for these lesions may be possible with the TMR 10 dose calculation algorithm. These results are consistent with our previous studies on phantom and model patients.^(17)^ Therefore, the tissue inhomogeneity effect might be a nonnegligible factor in the dose calculations for Gamma Knife radiosurgery.

Large differences in the dose statistics from the convolution and the TMR 10 algorithms were observed for the treatment targets in the close vicinity of the bone and/or air inhomogeneities. The differences could be further amplified by the differences in the treatment times, in some cases. The limitation of the water‐based TMR 10 algorithm is evident for these cases, and the use of the convolution algorithm for treatment planning dose calculation is desired for optimal dose calculation results.

## V. CONCLUSIONS

We have performed a comparative study on the dose calculation algorithms and the skull definition methods in the Leksell GammaPlan treatment planning system. Except for a few cases, all the results from the imaging skull definition method and the conventional measurement‐based skull definition method are similar. An averaged difference of 8.4% was observed between the treatment times from the convolution and the TMR 10 calculations for the same dose prescription and the same shot arrangements. Large differences in the dose statistics from the convolution and the TMR 10 algorithms were also observed for the treatment targets in the close vicinity of the bone and/or air inhomogeneities, indicating that the convolution algorithm might be a better option for the treatment planning dose calculations in these cases.

## Supporting information

Supplementary Material
