# Supplementary figures and images for: Gamma Knife radiosurgery with CT image‐based dose calculation
Source: J Appl Clin Med Phys. 2015 Nov 8;16(6):119–29. doi: 10.1120/jacmp.v16i6.5530 (PMC5691031; doi:10.1120/jacmp.v16i6.5530)

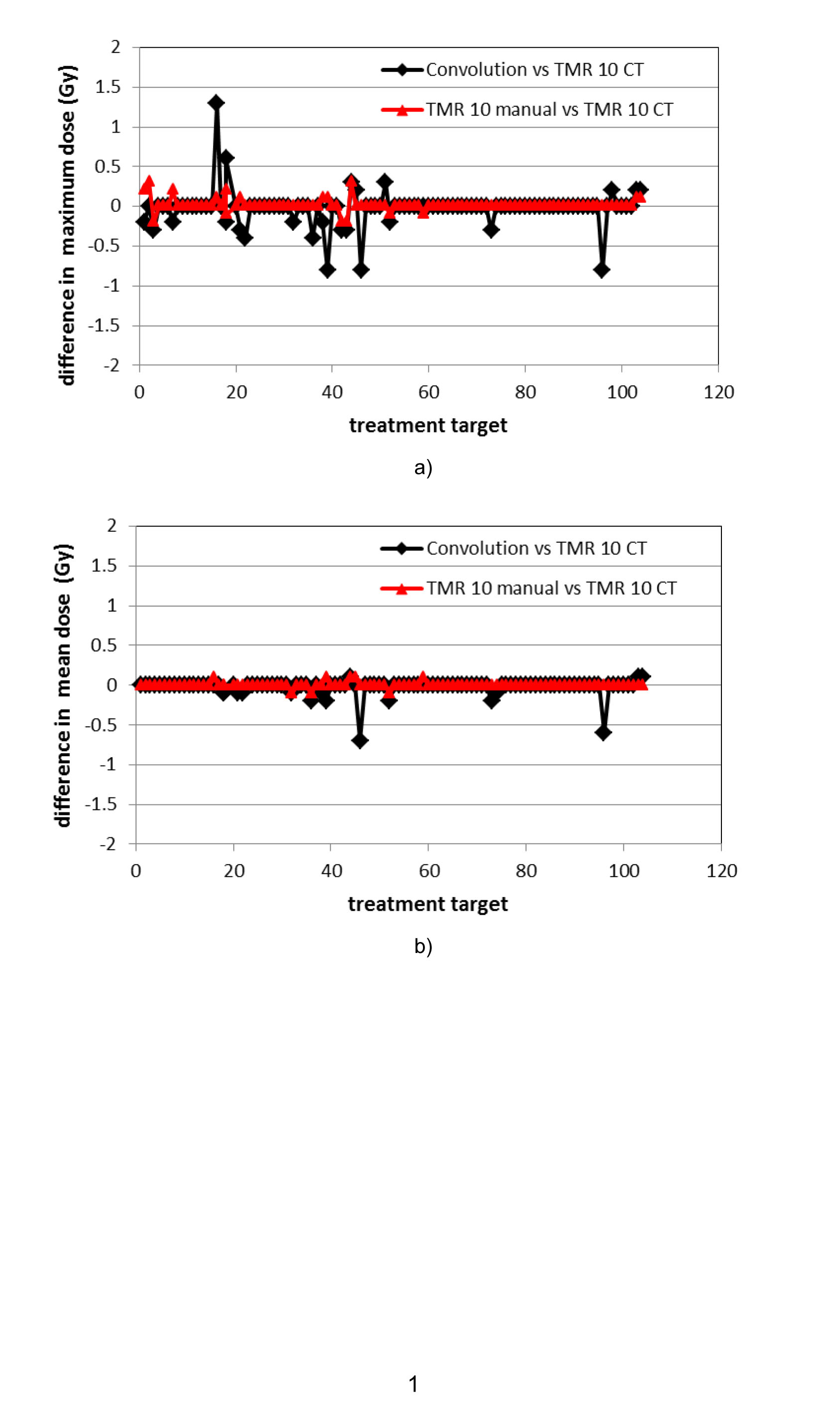

Supplement: Supplementary file 1 — Supplementary Material [file ACM2-16-119-s001.jpg]
